# Seabird diversity and biomass enhance cross-ecosystem nutrient subsidies

**DOI:** 10.1098/rspb.2022.0195

**Published:** 2022-05-11

**Authors:** Cassandra E. Benkwitt, Peter Carr, Shaun K. Wilson, Nicholas A. J. Graham

**Affiliations:** ^1^ Lancaster Environment Centre, Lancaster University, Lancaster LA1 4YQ, UK; ^2^ Institute of Zoology, Zoological Society of London, Outer Circle, Regent's Park, London NW1 4RY, UK; ^3^ Chagos Conservation Trust, 23 The Avenue, Sandy, Beds SG19 1ER, UK; ^4^ Marine Science Program, Department of Biodiversity Conservation and Attractions, Kensington, Western Australia, Australia; ^5^ Oceans Institute, University of Western Australia, Crawly, Western Australia, Australia

**Keywords:** biodiversity, coral reef, functional diversity, nutrient subsidy, restoration, seabird

## Abstract

Mobile consumers are key vectors of cross-ecosystem nutrients, yet have experienced population declines which threaten their ability to fill this role. Despite their importance and vulnerability, there is little information on how consumer biodiversity, in addition to biomass, influences the magnitude of nutrient subsidies. Here, we show that both biomass and diversity of seabirds enhanced the provisioning of nutrients across tropical islands and coral reefs, but their relative influence varied across systems. Seabird biomass was particularly important for terrestrial and near-shore subsidies and enhancing fish biomass, while seabird diversity was associated with nutrient subsidies further offshore. The positive effects of diversity were likely driven by high functional complementarity among seabird species in traits related to nutrient storage and provisioning. However, introduced rats and non-native vegetation reduced seabird biomass and diversity, with rats having a stronger effect on biomass and vegetation having a stronger effect on diversity. Accordingly, the restoration of cross-ecosystem nutrient flows provided by seabirds will likely be most successful when both stressors are removed, thus protecting both high biomass and diversity. Recognizing the importance of mobile consumer diversity and biomass, and their underlying drivers, is a necessary step to conserving these species and the ecosystem functions they provide.

## 1. Introduction

Nutrient flows across ecosystems can alter biomass, productivity, diversity, trophic interactions, food web complexity and stability in recipient ecosystems [1]. Mobile consumers play a unique and important role in transferring nutrients across ecosystem boundaries, as they actively redistribute nutrients across timescales, distances and gradients that are not possible with abiotic processes alone [2,3]. However, consumer-mediated nutrient transfer has declined worldwide due to human-induced environmental change, including overharvest, habitat fragmentation, invasive species and climate change [3,4]. Globally, the capacity of animals to move nutrients is estimated to be just 6% of pre-historic values, primarily due to reductions in population and body size of key vectors including seabirds, whales, salmon and terrestrial herbivores [5].

In addition to reducing the biomass of mobile consumers at a global scale, human activities are altering local patterns of biodiversity at an unprecedented rate via population-level declines and local extirpations [6–8]. These changes may result in reductions to nutrient transfer beyond those caused by biomass loss alone. Indeed, there is a positive relationship between species richness of fish and nutrient storage, provisioning and recycling within some aquatic and marine systems [9,10]. However, this work has focused on consumer-mediated nutrient dynamics *within* systems, rather than the transfer of nutrients *across* multiple ecosystems. Several recent reviews have posited that the diversity of mobile link species may similarly alter the magnitude, quality and extent of cross-ecosystem nutrient subsidies [11–13], but empirical tests of this relationship are lacking. Quantifying the relative and absolute effects of biomass and biodiversity on nutrient subsidies can inform effective ecosystem-based management strategies to maintain and restore natural nutrient pathways.

Seabirds are a key group of mobile link consumers, as they transfer and concentrate nutrients from their feeding grounds in the ocean to island and coastal regions where they roost and breed [14]. The majority of these nutrients are in the form of guano (excrement), which helps drive global nitrogen and phosphorus cycles, and contributes an estimated $500 million annually to ecosystem services worldwide [15–17]. At local scales, island and coastal ecosystems with abundant populations of breeding seabirds have enhanced productivity, biomass and ecosystem functioning compared to areas with few seabirds [18–22]. Although the importance of seabirds for providing nutrient subsidies is well-documented, most studies use grouped categories of high versus low seabird density so the relationship between biomass of seabirds and nutrient provisioning is generally unknown (but see [23,24]). Moreover, no studies have examined the effects of seabird diversity on their nutrient transfer and provisioning across ecosystems. Documenting these relationships is particularly urgent because seabirds are experiencing intense population declines and local extirpations due to human activities [25].

Here, we test how the biomass and biodiversity of seabirds breeding on islands influences the magnitude of their nutrient subsidies transferred to tropical islands and near-shore coral reefs. We first compare seabird-derived nitrogen values in terrestrial soil and leaves, and marine sponges, algae and fish across islands with a gradient of seabird biomass and species richness. Next, we test whether increased coral-reef fish biomass is associated with higher seabird biomass and/or diversity, which would indicate not only greater transfer of seabird-derived nutrients across multiple systems but also greater benefits. Finally, we examine the drivers of seabird biomass and biodiversity, then identify seabird species and functional traits most associated with areas of high seabird biomass, biodiversity, and nutrient transfer. Combined, these results can inform which management actions and outcomes should be a focus of seabird conservation efforts to restore consumer-derived nutrient subsidies to vulnerable tropical island and coral-reef ecosystems.

## 2. Methods

We conducted this study on and around 12 low-lying islands in the northern atolls of the Chagos Archipelago, Indian Ocean (5°50′ S, 72°00′ E) (electronic supplementary material, table S1). The entire region is characterized by minimal local human influence, as all islands are uninhabited and the surrounding waters are protected by a very large marine protected area [26]. However, there are remnants of previous human impacts on some islands, as non-native rats and coconut plantations were introduced to some islands several hundred years ago [27]. Both of these stressors are known to reduce seabird populations—introduced rats consume seabirds and seabird eggs, and seabirds avoid nesting or roosting on introduced coconut palms [20,22,25,28]. Of our 12 study islands, 6 have invasive rats and 6 are rat-free, and 8 have some non-native forest while 4 have fully intact native vegetation (electronic supplementary material, table S1).

### (a) Seabirds

The Chagos Archipelago supports 18 species of breeding seabird, with 15 species present on our study islands. Four species breed in globally significant numbers that trigger four internationally recognized Important Bird and Biodiversity Areas (IBAs) [29]. Approximately 4–5% of the total population of seabirds in the western Indian Ocean breed in the Archipelago [26,29]. We used breeding pair densities for these islands from [29], which is based on the maximum number of seabirds observed during surveys conducted between 2008 and 2018 and is the most up-to-date and comprehensive study of seabirds in the area.

We converted number of breeding pairs per island to biomass (kg ha^−1^) following the formula:

$$\;{\mathrm{Biomass}}_{i\;}=\;\overset{\;}{\underset{j}{\sum }}2\;\times \;{\mathrm{BreedingPairs}}_{ij\;}\times \;{\mathrm{Mass}}_{j}\;\times \;{\mathrm{BreedingSeason}}_{j}\;\times \;{\mathrm{Area}}_{i}^{-1},$$

where the total annual biomass on each island (*i*) is the sum across all species (*j*) of 2 (birds per breeding pair) multiplied by the number of breeding pairs, the species-specific average adult mass [30] and the species-specific length of the breeding season in the Chagos Archipelago (proportion of time per year on island), divided by the island area.

We used species richness (number of species/island) as our measure of seabird diversity. We also measured functional diversity based on a number of traits that are relevant to nutrient transfer [31]—body size, breeding duration, breeding habitat, foraging habitat, foraging trip duration and diet (electronic supplementary material, table S2). To calculate functional diversity, we used these traits in a multivariate analysis based on pairwise functional distances between species following standard methods (see 'Statistical analyses' below) [32,33]. We took both a species-based and trait-based approach because active management initiatives for seabirds typically focus on specific species [34], while functional traits may better predict ecosystem function, including nutrient cycling and transfer [31,35,36].

Two species (*Phaethon lepturus* and *Sterna dougallii*) were extremely rare both in the recent seabird surveys and in historical records of seabirds in the Archipelago, with only 7 total breeding pairs (no more than 2 per island) of *P. lepturus* and 6 total breeding pairs (less than or equal to 3 breeding pairs on any one island) of *S. dougalli* recorded during recent surveys. Therefore, we ran all calculations and analyses with and without these two rare species, which presumably have only a small influence on nutrient cycling in the region, to ensure that they were not disproportionately affecting the results. Excluding these species had little effect on seabird biomass or species richness (correlation of island-level seabird biomass including all species versus excluding these species = 0.99; correlation of island-level seabird richness including versus excluding these species = 0.96) or on any results, so we report only on analyses with all species in the manuscript, but all analyses are available at github.com/cbenkwitt/seabird-diversity-nutrients.

### (b) Nutrients

We quantified the magnitude of nutrient subsidy provision from seabirds by measuring the ratio of isotopic nitrogen ^15^N to ^14^N relative to the ratio in standard reference material of atmospheric nitrogen (expressed as *δ*^15^N) in both terrestrial and marine samples. *δ*^15^N provides a reliable tracer of seabird-derived nutrients, with higher values indicating a higher proportion of seabird-derived nutrients compared to local nutrient sources, in part because seabirds feed at a relatively high trophic level [18,20,37–39]. Although other sources and processes can also alter *δ*^15^N (e.g. pollution, denitrification), seabirds are the primary drivers of *δ*^15^N shifts in this system and other remote tropical islands, evidenced in part by high congruence between *δ*^15^N values from tropical seabird guano (mean *δ*^15^N approx. 10–18%) [22,37] with terrestrial samples from areas with abundant seabirds (mean *δ*^15^N approx. 8–16%) [20,22,40]. Furthermore, we used *δ*^15^N in a comparative manner across islands with differing seabird populations and assumed other sources and processes that influence *δ*^15^N were similar across our study islands given their similarity in characteristics including climate and distance from human populations.

We collected ten replicates of each of six sample types (soil, leaves, sponge, macroalgae, turf algae and fish) from each island in March 2015. We collected topsoil inland of the coastal vegetation boundary, and new growth leaves of *Scaveola taccada* along the beach. We sampled sponges (*Spheciospongia* sp.) and macroalgae (*Halimeda* sp.) along the reef flat at approximately 1 m depth and 100 m from shore, and collected turf algae and herbivorous damselfish (*Plectroglyphidodon lacrymatus*) along the reef crest at approximately 3 m depth and 230 m from shore. Logistical and weather constraints prevented fish sampling at one of the islands (Nelson's). We took a sample of dorsal white muscle, with skin removed, from each fish. Immediately following collection, all samples were dried at 60°C for at least 24 h and powdered with a pestle and mortar. To remove calcareous matter, we added a few drops of 1 M hydrochloric acid to all soil, sponge, and algae samples in silver cups until effervescence stopped. *δ*^15^N values from a subset of sample analysed with and without this treatment had correlation coefficients between 0.9 and 0.99, indicating a minimal effect of acid washing on *δ*^15^N. We conducted stable isotope analysis using a Finnigan MAT Delta Plus Isotope Ratio Mass Spectrometer coupled with a Costech 4010 Elemental Analyzer at the University of Windsor, Canada. We ran samples in duplicate or triplicate to ensure precision of readings. Readings were within 0.3% for soil and 0.1% for all other samples based on B2153 and USGS40 internal standards, respectively.

### (c) Fish biomass

We quantified fish biomass around each island along 4 replicate 30 m transects. Along each transect, one observer recorded the species and size (total length to the nearest cm) of all non-cryptic, diurnal fishes greater than 8 cm total length. We counted large and mobile fishes along a 5 m-wide belt, and damselfishes in a 2 m wide belt during a second pass of the same transect. We used published length-weight relationships to convert fish counts and sizes to biomass [41]. Because they can be strong drivers of coral-reef fish biomass, we also measured live coral cover and structural complexity along each transect [42,43]. We quantified coral cover using the line-intercept method, and structural complexity using a standard scale ranging from 0 (no vertical relief) to 5 (exceptionally complex).

### (d) Statistical analyses

We used a series of multilevel Bayesian models to test the relative and absolute effects of (1) seabird biomass and diversity on the magnitude of their nutrient subsidies on islands and coral reefs, (2) seabird biomass and diversity on coral-reef fish biomass and (3) introduced rats and non-native forest on seabird biomass and diversity. We found no evidence for multicollinearity between explanatory variables within each model (all variance inflation factors [VIFs] less than 4), so we could reliably partition the effects of the explanatory variables within each model. To compare the relative effect sizes of predictors, we centered and standardized continuous predictors in all models. To estimate absolute effect sizes, we re-ran all models with centered, non-standardized predictors.

For (1), we used *δ*^15^N values for soil, leaves, sponges, macroalgae, turf algae and fish, which indicate the magnitude of seabird-derived nutrient subsidies, as response variables in a multivariate model. Seabird biomass and species richness were explanatory variables, with island included as a correlated group-level effect to account for the inclusion of multiple replicates per island and different island attributes.

To determine the effects of seabird biomass and diversity on total coral-reef fish biomass (2), we analysed log-transformed fish biomass (per transect) as a function of seabird biomass and species richness, again with island as a group-level effect to account for multiple transects per island. We included structural complexity and coral cover as additional covariates in this model to determine the effects of seabirds on fish biomass after accounting for these other important predictors [42,43].

To help determine the drivers of seabird biomass and diversity (3), we ran a multivariate model with seabird biomass and species richness as response variables and introduced rat status (rat-infested versus rat-free) and percentage non-native forest as explanatory variables. We did not include island in this model because there was only one measurement per island; instead we included atoll as a correlated group-level effect to account for the spatial distribution of islands across multiple atolls. Models for seabird biomass included seabird species richness as an additional explanatory variable because of the predicted positive relationship between biodiversity and ecosystem function [44,45]. Models for species richness included island size as an additional explanatory variable because, unlike for biomass, size was not already accounted for in the response, and there is a well-documented positive relationship between species richness and area, including for seabirds [46].

For all models, we log-transformed seabird biomass because preliminary analyses and graphical checks indicated a nonlinear relationship between seabird biomass with *δ*^15^N values and fish biomass. We ran models for 4 chains, each with 3000 iterations and a warm-up of 1000 iterations. We assessed model convergence and fit using posterior predictive checks, traceplots, and the Gelman-Ruban convergence diagnostic (R-hat) [47]. All models were run in R and implemented in STAN using the package *brms* [48,49].

We conducted a principal component analysis (PCA) on the scaled density of breeding pairs to examine variation in seabird community composition among islands and identify seabird species driving these patterns. We included total seabird biomass, species richness, median *δ*^15^N of each sample type and median fish biomass on each island as supplemental variables, which enabled us to relate these variables of interest to specific seabird species and communities. To further quantify which seabird species were most associated with high seabird biomass and diversity, we examined the correlations between density of each species per island with total seabird biomass and species richness per island. We used the packages *FactoMineR* and *factoextra* to conduct and visualize the PCA [50,51].

Finally, we created a multi-dimensional functional space by conducting a principal coordinate analysis (PCA) on the distance between seabird species based on functional traits [33]. We gave each trait (electronic supplementary material, table S2) equal weight in calculating the distance matrix, and used Gower distance because it can handle a mix of continuous, ordinal, and categorical traits. The highest quality functional space had 3-dimensions, so we used this to visualize the relationships between species in functional space, with species that appear closer together in the PCA being more functionally similar, and those that are farther apart more functionally distinct. We also determined the correlations between the 3 PCA axes and functional traits (electronic supplementary material, table S2). We calculated functional richness of each island as the proportion of functional space filled by the convex hull of each island's species assemblage. Functional richness was highly correlated with species richness (corr = 0.94), so was not used in formal analyses because it did not provide any additional information. We conducted all functional diversity analyses using the package *mFD* [32].

All analyses were conducted in R v. 3.6.1 [52], and all data and code are publicly available at github.com/cbenkwitt/seabird-diversity-nutrients.

## 3. Results

### (a) Seabird biomass and diversity enhance nutrient subsidies

**Figure 1.**
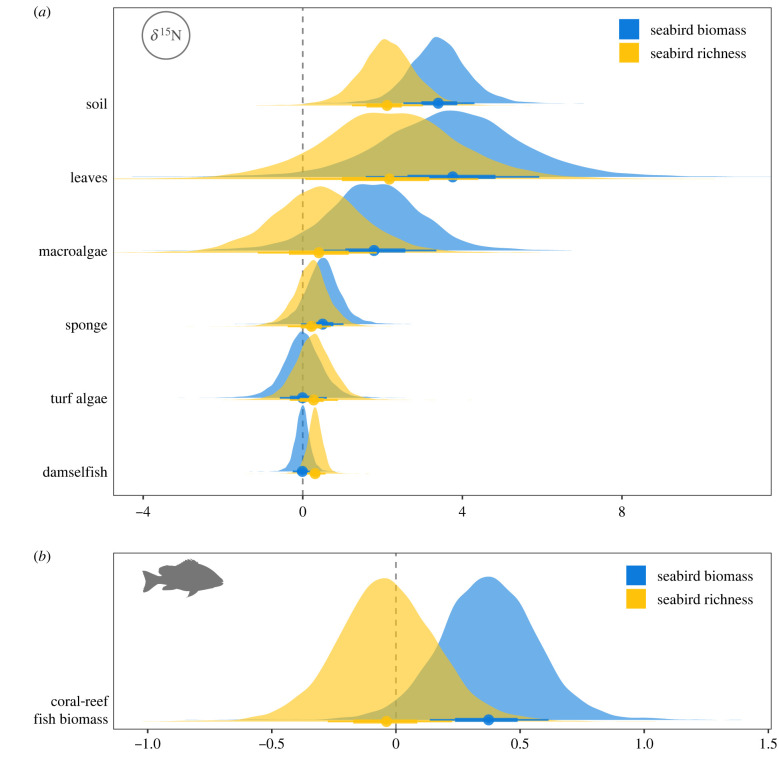
Posterior distributions for the standardized effects of seabird biomass (log[kg ha^−1^]) and diversity (species richness) on (*a*) *δ*^15^N values of soil, leaves, macroalgae, sponge, turf algae and damselfish and (*b*) total coral-reef fish biomass (log[kg ha^−1^]). Points indicate median estimates and bars represent 50 and 80% highest posterior density intervals (HPDIs). (Online version in colour.)

Biomass and diversity of breeding seabirds on tropical islands enhanced nutrient subsidies in terrestrial and marine systems, with consistently positive estimates for the effect of both biomass and diversity on *δ*^15^N values across sample types (figure 1*a*; electronic supplementary material, table S3). Although the relative effect sizes of biomass and species richness were generally similar, as demonstrated by overlapping posterior distributions for all samples, biomass had a stronger effect on *δ*^15^N values of soil, leaves, macroalgae, and sponges, whereas species diversity had a stronger effect on turf algae and damselfish, which were collected farthest offshore (figure 1*a*). In terms of absolute effect sizes, after accounting for species richness, a doubling of seabird biomass resulted in an estimated increase in median *δ*^15^N of 0.62, 0.70, 0.34 and 0.10 for soil, leaves, macroalgae, and sponges respectively, while there were negligible increases in *δ*^15^N values of turf algae and fish (electronic supplementary material, figure S1 and table S3). By contrast to this log-linear increase of *δ*^15^N with increasing seabird biomass, *δ*^15^N increased linearly with increasing species richness. After accounting for seabird biomass, for each additional species of seabird, *δ*^15^N increased by an estimated 0.85, 1.18, 0.17, 0.06, 0.09 and 0.13 for soil, leaves, macroalgae, sponges, turf algae and fish, respectively (electronic supplementary material, figure S1 and table S3).

### (b) Seabird biomass enhances fish biomass

Seabird biomass, but not seabird diversity, had a positive effect on fish biomass (figure 1*b*; standardized estimates = 0.37, −0.04; 95% HPDI = −0.01 to 0.75, −0.44 to 0.37, respectively). After accounting for coral cover, reef structural complexity and seabird species richness, median fish biomass increased by a factor of 1.07 for each doubling in seabird biomass (95% HPDI = 1.00 to 1.15) (electronic supplementary material, figure S2).

### (c) Introduced rats and non-native vegetation reduce seabird biomass and diversity

**Figure 2.**
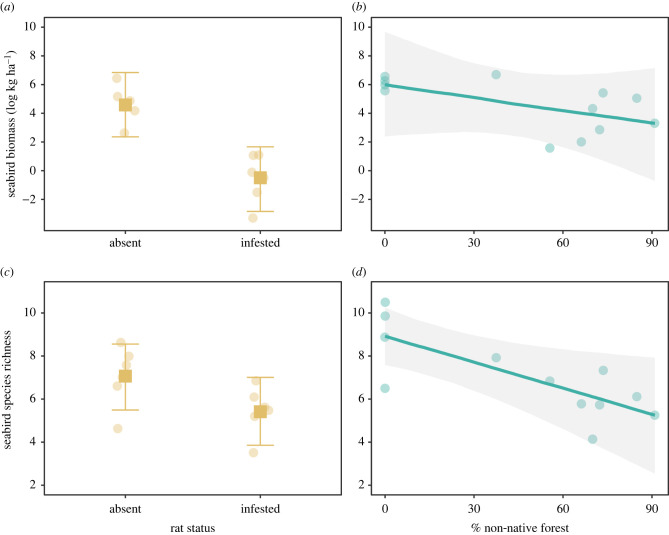
Conditional effects of introduced rats and non-native vegetation on (*a*,*b*) seabird biomass (log[kg ha^−1^]) and (*c*,*d*) diversity (species richness). (*a*,*c*) Circles represent partialized residuals, squares represent median estimates and error bars represent 75% confidence intervals from multilevel Bayesian models. (*b*,*d*) Points represent partialized residuals, lines represent predicted fits and shading represents 75% confidence intervals from multilevel Bayesian models. (Online version in colour.)

Introduced rats and non-native vegetation both negatively influenced seabird biomass and seabird richness, but the magnitude of their effects on biomass and diversity differed (figure 2). The presence of introduced rats on islands had the strongest negative effect on seabird biomass, whereas percentage of non-native vegetation had the strongest negative effect on seabird diversity (electronic supplementary material, figure S3). After accounting for non-native vegetation and seabird richness, median seabird biomass was an estimated 162.39 times higher on rat-free compared to rat-infested islands (electronic supplementary material, table S3). By contrast, seabird species richness declined by approximately one species for each 25% increase in non-native vegetation, after accounting for rat status and island size (electronic supplementary material, table S3).

### (d) Seabird species associated with high biomass and diversity are functionally diverse

**Figure 3.**
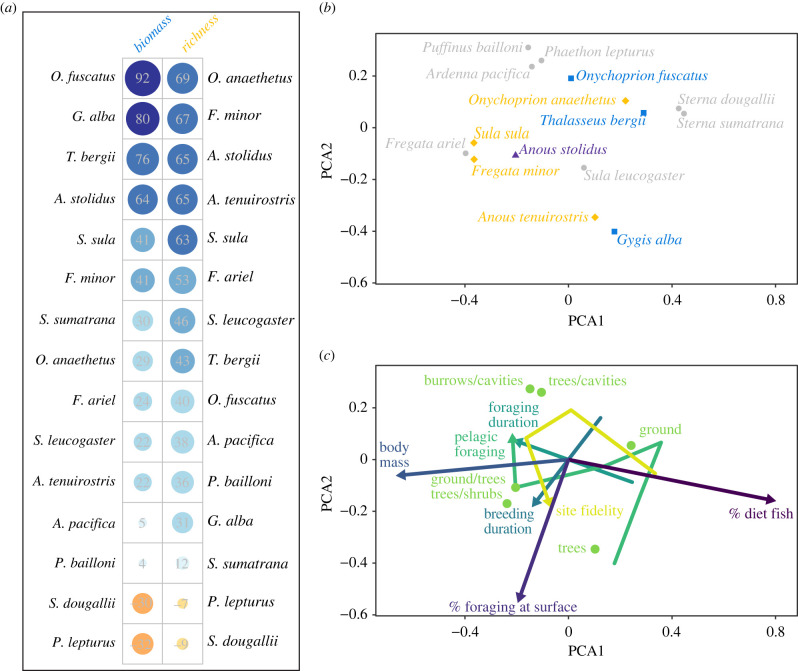
Species and functional characteristics of seabird communities. (*a*) Correlations between breeding pair density of each seabird species with total seabird biomass and seabird species richness. Grey values are correlations expressed as percentages. (*b*) First two axes from a trait-based principal coordinates analysis (PCA) of seabird species in functional space. Shape and text colour represent species with strong correlations with total biomass (blue square), species richness (yellow diamond), both (purple triangle) or none (grey circle) from panel (*a*). (*c*) Overlay of functional traits on the same functional space axes as in (*b*). Ordinal and numeric traits are shown with arrows and text, with nonlinear arrows occurring for ordinal traits with greater than two categories. Circles show average trait position for the categorical trait nesting habitat. See electronic supplementary material for tables of functional traits and their correlations with PCA axes, and plots of PCA3. (Online version in colour.)

Species representing a range of taxonomic and functional groups drove patterns in seabird biomass and diversity (figure 3). *Onychoprion fuscatus* (sooty tern), *Gygis alba* (white tern), *Thalasseus bergii* (crested tern), and *Anous stolidus* (brown noddy) were most correlated with total seabird biomass, while *Sula sula* (red-footed booby), *A. stolidus* (brown noddy), *A. tenuirostris* (lesser noddy), *O. anaethetus* (bridled tern) and *Fregata minor* (lesser frigatebird) were most correlated with species richness (figure 3*a*). The latter five species were also best represented by the first dimension of a separate PCA, which further corroborated the links between seabird biomass, diversity, nutrient transfer and ecosystem function identified in the Bayesian analyses (electronic supplementary material, figure S4 and table S5). While some of the seabird species driving community patterns had high overall density and biomass (e.g. *O. fuscatus*, *A. tenuirostris*, *S. sula*), others had relatively low density and biomass (e.g. *G. alba*, *T. bergii*, *O. aneathetus*, *F. minor*).

In trait space, seabird species separated by a combination of foraging and breeding-related traits, with foraging duration, neritic versus pelagic foraging habitat, diet, breeding site fidelity and body mass driving the first dimension of the PCA. By contrast, breeding duration, nesting habitat and surface versus subsurface foraging habitat were most correlated with PCA2, while body mass and surface foraging habitat were most correlated with PCA3 (figure 3*b*,*c*; electronic supplementary material, figure S5 and table S6). Importantly, the key seabird species associated with seabird biomass, diversity and nutrient transfer were functionally diverse, as they occupied a wide breadth of positions along all PCA axes (figure 3*b*,*c*; electronic supplementary material figure S5).

## 4. Discussion

Understanding the drivers of cross-ecosystem nutrient subsidies provides basic knowledge of ecosystem structure and function that can inform management actions to revive lost nutrient pathways. Here, we show that biomass and biodiversity of a key group of mobile link organisms—seabirds—were important predictors of nutrient transfer to tropical island and near-shore coral-reef ecosystems. The relative effects of biomass and biodiversity varied across terrestrial and marine systems depending on the measured ecosystem response (nutrient uptake or fish biomass), suggesting that the restoration of natural nutrient pathways across multiple ecosystems will be most successful when both high biomass and high diversity of seabirds are conserved. Because the presence of introduced rats was the primary driver of seabird biomass while the amount of introduced vegetation was a stronger driver of seabird diversity, removing both rats and non-native vegetation is likely essential to have a fully functioning seabird-driven system. Overall, conserving species and functional diversity of mobile consumers, in addition to biomass, is essential to maintaining and restoring ecosystem services across multiple systems.

The log-linear increase in seabird-derived nitrogen in both terrestrial and marine samples with increasing biomass of tropical seabirds indicates a saturating relationship between nitrogen provisioning and seabird biomass. *δ*^15^N values in terrestrial plants and invertebrates exhibit similar saturating relationships with increasing biomass of salmon, which transport nutrients from oceanic to aquatic and terrestrial systems via spawning migrations [53]. Few other studies have examined how seabird-derived nutrient subsidies vary across a gradient of seabird density or biomass, and those that have been conducted document different relationships. For example, nitrogen values in marine algae are only elevated near Baltic Sea islands with high densities of cormorant nests, compared to nearby islands with low nest density, recently abandoned nests or no nests [54]. These results suggest nutrient subsidies from seabirds are only transferred from island to coastal systems once densities reach a critical threshold. By contrast, there is a linear relationship between increasing breeding pairs of penguins and the spatial extent of nitrogen inputs in terrestrial systems in Antarctica [24]. Combined, these findings suggest that the transfer of nutrients by mobile consumers is dependent on consumer density or biomass, but the nature of this relationship is variable across systems and species. Thus, quantifying the shape of this relationship in different contexts is an important avenue of future research.

Seabird biomass not only enhanced cross-ecosystem nutrient transfer but was also an important predictor of fish biomass. This result was somewhat surprising given that seabird biomass had a negligible effect on *δ*^15^N in damselfish and the turf algae that they consume. However, herbivorous damselfish comprise only a small proportion of total fish biomass, and it is possible that other feeding groups have nutrient levels that are more affected by seabird biomass. Indeed, seabird biomass was a stronger predictor of nutrient levels in macroalgae and sponges, so these differences may be propagated up the food chain. Additional basal resources may similarly be more affected by seabird biomass. For example, plankton is an important source of energy and nutrition on many coral reefs, and planktivores can drive patterns in overall fish assemblages due to their high relative abundance [55,56]. Overall, the strong positive effect of seabird biomass on fish biomass is consistent with previous work demonstrating higher fish growth, productivity and biomass around rat-free islands with abundant seabirds compared to rat-infested islands with few seabirds [20,21,57,58].

The relationship between cross-ecosystem nutrient subsidies and biodiversity has primarily been studied from the perspective of how nutrient inputs influence diversity in recipient ecosystems [21,59–64]. Here, we take the opposite approach, demonstrating that the diversity of mobile consumers increases the magnitude of their subsidies, even after accounting for biomass. Seabird diversity had particularly strong absolute effects on terrestrial nutrient values, and particularly strong relative effects on marine turf algae and fish. Indeed, for turf algae and damselfish, seabird diversity, not biomass, was an important predictor of enhanced *δ*^15^N values, although the absolute effect size was small. There are multiple non-mutually exclusive explanations for this finding. First, turf algae and damselfish were the two organisms collected farthest offshore, suggesting that seabird diversity may increase the spatial extent of their nutrient subsidies. Because seabird guano can enter the marine environment either via surface run-off or percolation from islands, or through direct deposition by flying seabirds [37], having higher seabird diversity, including species with different nesting, defecating and foraging behaviours, may increase the potential pathways and spatial distribution of seabird-derived nutrients. Second, turf algae is a conglomeration of different algal and microbial species, all of which are likely to have different nutrient requirements and are then consumed and assimilated by territorial damselfish [65]. Thus, different species within the turf algal matrix may be able to better capitalize on slightly different nutrients provided by various seabird species. Regardless of the exact mechanism, the positive effects of seabird diversity on nutrient values of turf algae and fish, as well as on other organisms, provide empirical evidence for the idea that higher biodiversity of mobile link species lead to increased magnitude, quality and extent of cross-ecosystem nutrient subsidies [11–13].

More broadly, we documented a positive relationship between consumer biodiversity and an important ecosystem function; cross-ecosystem nutrient transfer by mobile organisms. Although the effects of biodiversity on cross-ecosystem nutrient transfer have previously been overlooked, our results are consistent with a large body of empirical and theoretical work demonstrating positive relationships between biodiversity and ecosystem functioning (BEF) in general [44,45]. Our results also complement prior work on the effects of consumer diversity on other forms of cross-ecosystem connectivity, including seed dispersal by dung beetles [66] and carbon capture by sessile mussels [67]. Similarly, there are positive effects of consumer diversity on nutrient provisioning and recycling within systems [9,10,68], although these biodiversity benefits are sometimes outweighed by the effects of biomass [10,68]. Strong effects of biodiversity on ecosystem function, including nutrient provisioning and cycling, are predicted to occur where there is a high degree of complementarity (i.e. low redundancy) among species in terms of their functional traits, behaviour or fluctuations in population size [45,69]. Our findings match this prediction, as species and functional diversity were highly correlated. Moreover, the top species associated with high seabird biomass, diversity, and nutrient transfer represented almost the entire range of adult body masses, nesting habitats, foraging habitats and foraging durations, all of which are predicted to be important in driving nutrient storage and transport [31,70,71]. In addition to variation among species or functional groups, there can be intraspecific differences in traits such as diet, stoichiometry, movement, and behaviour, all of which can strongly influence nutrient transfer [2,72,73]. Such intraspecific variation may be particularly relevant for seabirds, which display seasonal-, location-, sex- or age-related differences in diet and foraging behaviour [74,75]. Thus, including intraspecific diversity may further increase our ability to predict the magnitude of cross-ecosystem nutrient subsidies.

Finally, the relationships between introduced rats and non-native vegetation with seabird biomass and diversity shed additional light on the dynamics of this system and on necessary management actions to restore cross-ecosystem nutrient subsidies. Our results suggest that removing both rats and coconut palms is necessary to restore high seabird biomass and high seabird diversity, and thus high transfer of nutrients across ecosystems. The negative effects of introduced rats on seabirds is well-documented, and there is convincing evidence that seabird populations and their associated nutrient subsidies benefit from rat eradication [40,76,77]. In addition to removing rats, the importance of removing coconut plantations and restoring native vegetation in benefitting tropical seabird populations is increasingly recognized [28,78]. Here we show that vegetation management is likely a key component of tropical island management, due to the strong negative effects of non-native vegetation on not only seabird biomass but also (and perhaps more so) on seabird diversity. Indeed, several of the seabird species that contributed to high seabird diversity only bred on rat-free islands with intact native vegetation (e.g. bridled tern, lesser frigatebird), and therefore high seabird diversity may serve as an indicator of intact, seabird-driven island ecosystems.

Seabird management plans are increasingly focused on not only enhancing seabird populations but also on restoring nutrient subsidies and associated ecosystem functioning [34,79]. Such efforts may result in particularly large conservation gains on islands and near-shore coral reefs, which often rely on allochthonous nutrient inputs. For example, the importance of mobile consumers, including seabirds, sharks and fish, in providing, aggregating and redistributing nutrient and energy on coral reefs is increasingly recognized [20,37,80–82]. These consumer-derived nutrient subsidies, in turn, can bolster biomass, productivity and ecosystem functioning of coral-reef fishes [20,21], boost coral growth rates [81,83] and may speed reef recovery following climate-induced bleaching events [57]. Due to the benefits of resource subsidies to coral reefs, explicitly focusing on protecting and enhancing mobile link species may enhance the resilience of these threatened ecosystems [84]. Here, we show that conserving both biomass and diversity of seabirds is critical to maintain important seabird-derived nutrient subsidies to tropical islands and coral reefs, and to achieve this combination of high seabird biomass and diversity both introduced rats and non-native vegetation must be managed. More broadly, quantifying the benefits of biomass, biodiversity and specific species for cross-ecosystem nutrient transfer can inform broader efforts to restore lost natural nutrient pathways, and thus maintain ecosystem functioning despite human-caused environmental changes.

## Data Availability

All data and code are available on Github, accessible at: https://github.com/cbenkwitt/seabird-diversity-nutrients.
